# Intrapancreatic Accessory Spleen: Two Case Reports of a Rare Entity

**DOI:** 10.7759/cureus.8797

**Published:** 2020-06-24

**Authors:** Stylianos Kykalos, Nikolaos Machairas, Ernesto P Molmenti, Georgios Sotiropoulos

**Affiliations:** 1 Second Department of Propaedeutic Surgery, Laiko General Hospital, National and Kapodistrian University of Athens Medical School, Athens, GRC; 2 Hepato-Pancreatico-Biliary Surgery, Royal Free Hospital, London, GBR; 3 Department of Surgery, North Shore University Hospital, Manhasset, USA

**Keywords:** pancreatic tumor, pancreas lesion, accessory spleen, intrapancreatic spleen, neuroendocrine carcinoma

## Abstract

Intrapancreatic accessory splenic tissue constitutes a very unusual anatomical variation. It is encountered mostly in the splenic hilum or within the pancreatic tail. Given the diagnostic difficulty in excluding a pancreatic malignancy, a distal pancreatectomy is usually performed. We herein report two cases of intrapancreatic accessory spleen. The first patient presented with left upper quadrant abdominal pain radiating to the back, caused by a 2-cm focal lesion in the pancreatic tail. The second patient underwent a distal pancreatectomy due to a postsplenectomy symptomatic pseudocyst that could not be treated conservatively. In both cases, the histopathological examination of the specimens revealed a 2-cm accessory spleen within the pancreatic tail. Intra and peripancreatic spleens represent 10-16% of all accessory spleens, and their sizes range from a few millimeters up to 2-3 cm. CT, MRI, and nuclear scintigraphy are all useful in establishing the diagnosis. It is occasionally difficult to differentiate accessory spleens from hypervascular pancreatic neoplasms, metastatic lesions, or splenic hilar lymphadenopathy. The surgical resection of an intrapancreatic spleen is only indicated in the case of diagnostic uncertainty or spleen-related hemato-oncological conditions such as immune thrombocytopenia (ITP).

## Introduction

Distal pancreatic tumors are characterized by their non-specific clinical presentation and late diagnosis. Surgical removal is indicated in malignant cases or in selected lesions such as hormonally active neuroendocrine tumors. Curative resections can be achieved only in 10% of malignant cases [1] since most prove to be metastatic or locally advanced by the time of detection.

Accessory splenic tissue is most commonly encountered in the splenic hilum, adjacent to or even within the pancreatic tail. Intrapancreatic accessory splenic tissue, frequently diagnosed as a “pancreatic tumor,” constitutes a very unusual anatomical variation [2,3]. Although the surgical resection of an intrapancreatic spleen is generally not indicated, the diagnostic difficulty in excluding a pancreatic malignancy often leads to a distal pancreatectomy. We report two cases of intrapancreatic accessory spleens resected at our institution within a one-year period.

## Case presentation

Case report 1

**Figure 1:**
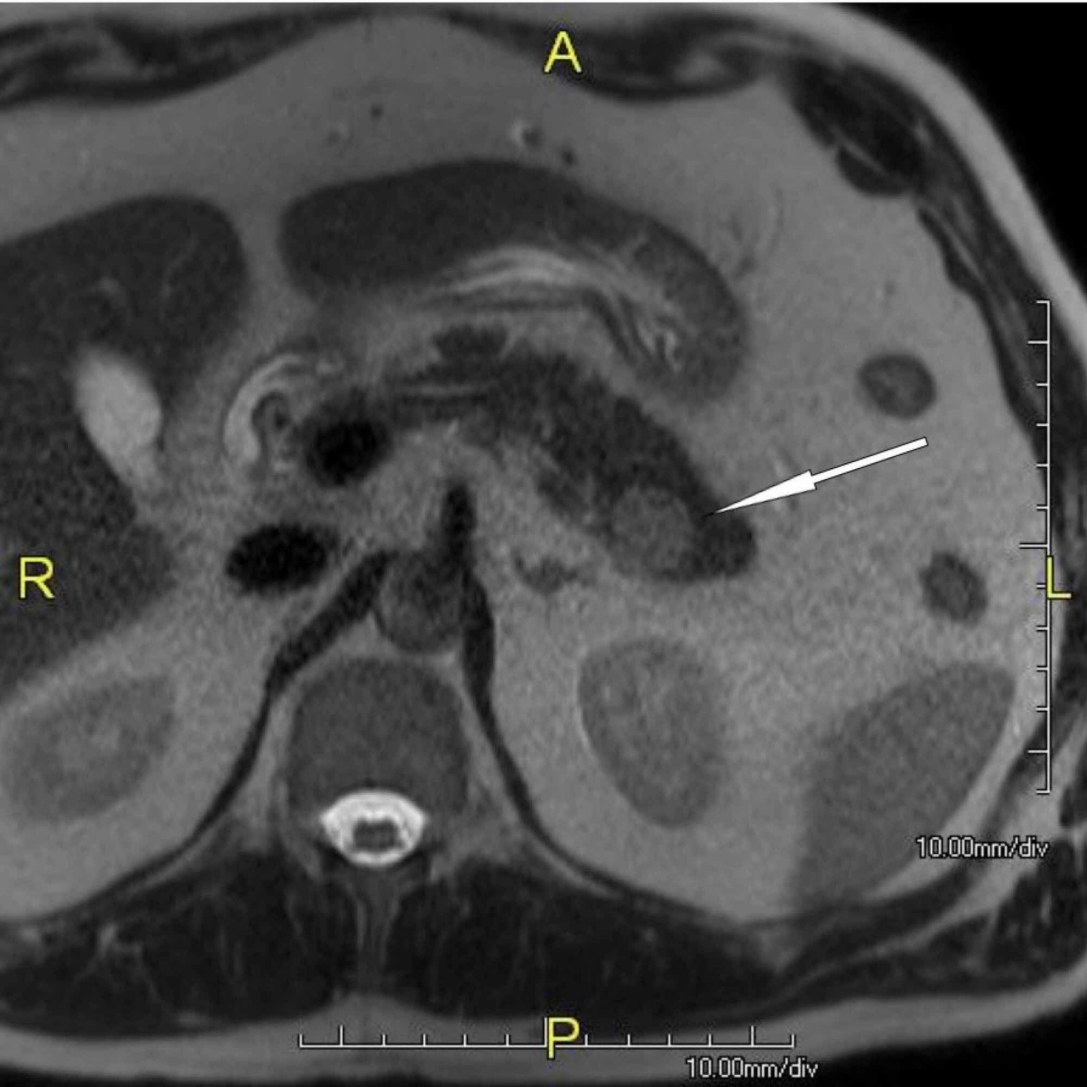
MRI of the 2-cm tumor in the pancreatic tail (intrapancreatic spleen) Intrapancreatic accessory spleen (arrow) on axial, fat-saturated, T2-weighted MR image, which shows high signal intensity compared to pancreas MRI: magnetic resonance imaging

A 54-year-old Caucasian male presented with left upper quadrant abdominal pain radiating to the back. He had no significant past medical history and laboratory tests including serum amylase, lipase, carcinoembryonic antigen (CEA), and carbohydrate antigen 19-9 (CA 19-9) were within normal range. Abdominal sonography revealed a 2-cm focal lesion in the pancreatic tail. MRI confirmed the findings (Figure 1).

There was no evidence of extrapancreatic spread. CT-guided fine-needle aspiration (FNA) was negative for malignancy. A spleen-preserving open distal pancreatectomy was undertaken. Histopathological examination of the specimen showed a 2-cm accessory spleen within the pancreatic tail. The postoperative course was complicated by a pancreatic fistula that resolved with percutaneous drainage. The patient was discharged three weeks after the operation.

Case report 2

**Figure 2:**
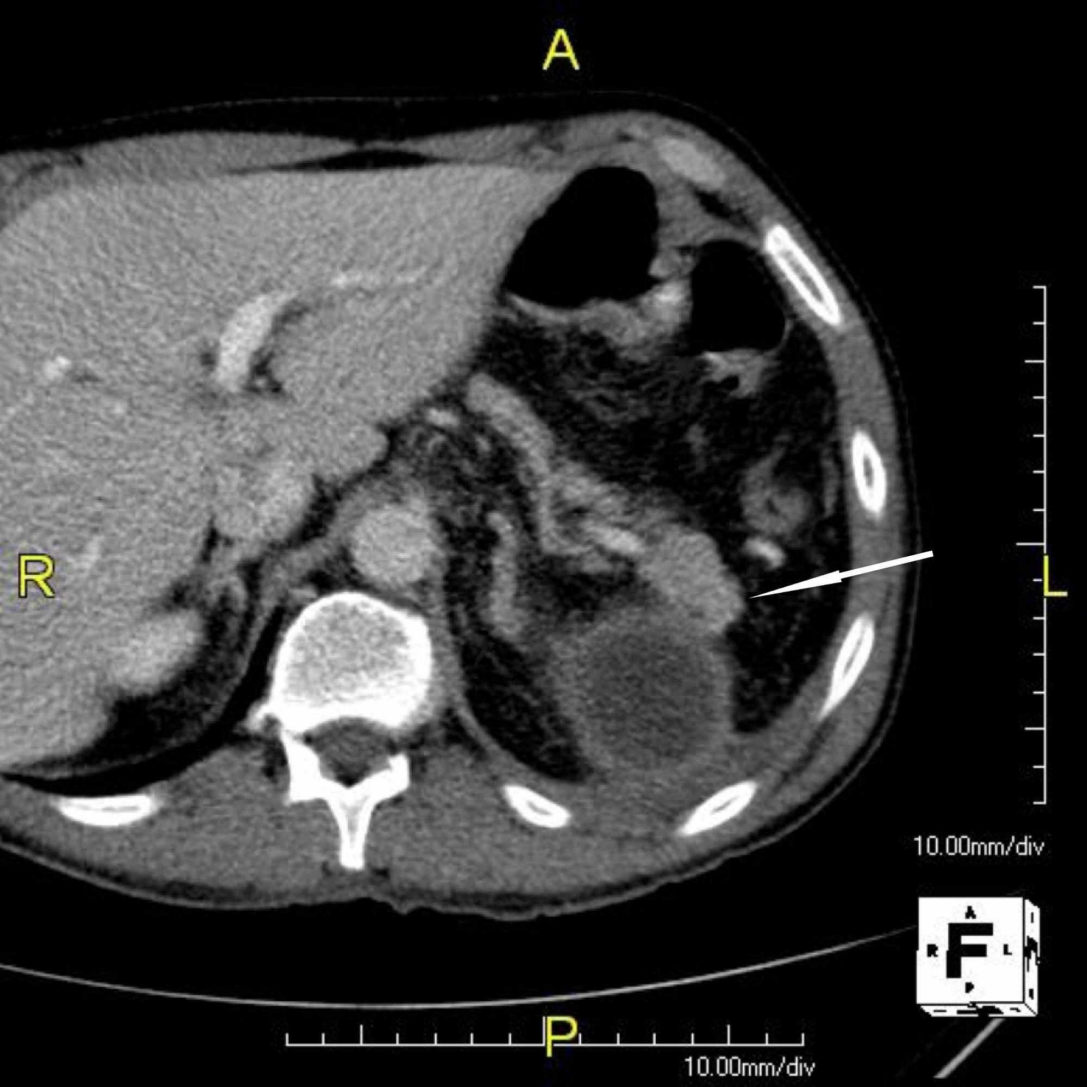
Contrast-enhanced axial CT image obtained during portal venous phase The main finding of the postsplenectomy pancreatic pseudocyst. The presence of an intrapancreatic accessory spleen (arrow) can be speculated in the tail of the pancreas CT: computed tomography

A 53-year-old Caucasian male with a traumatic rupture of the spleen underwent an emergency splenectomy complicated by a 7 x 6-cm pancreatic pseudocyst (identified by ultrasonographic and CT imaging, Figure 2).

**Figure 3:**
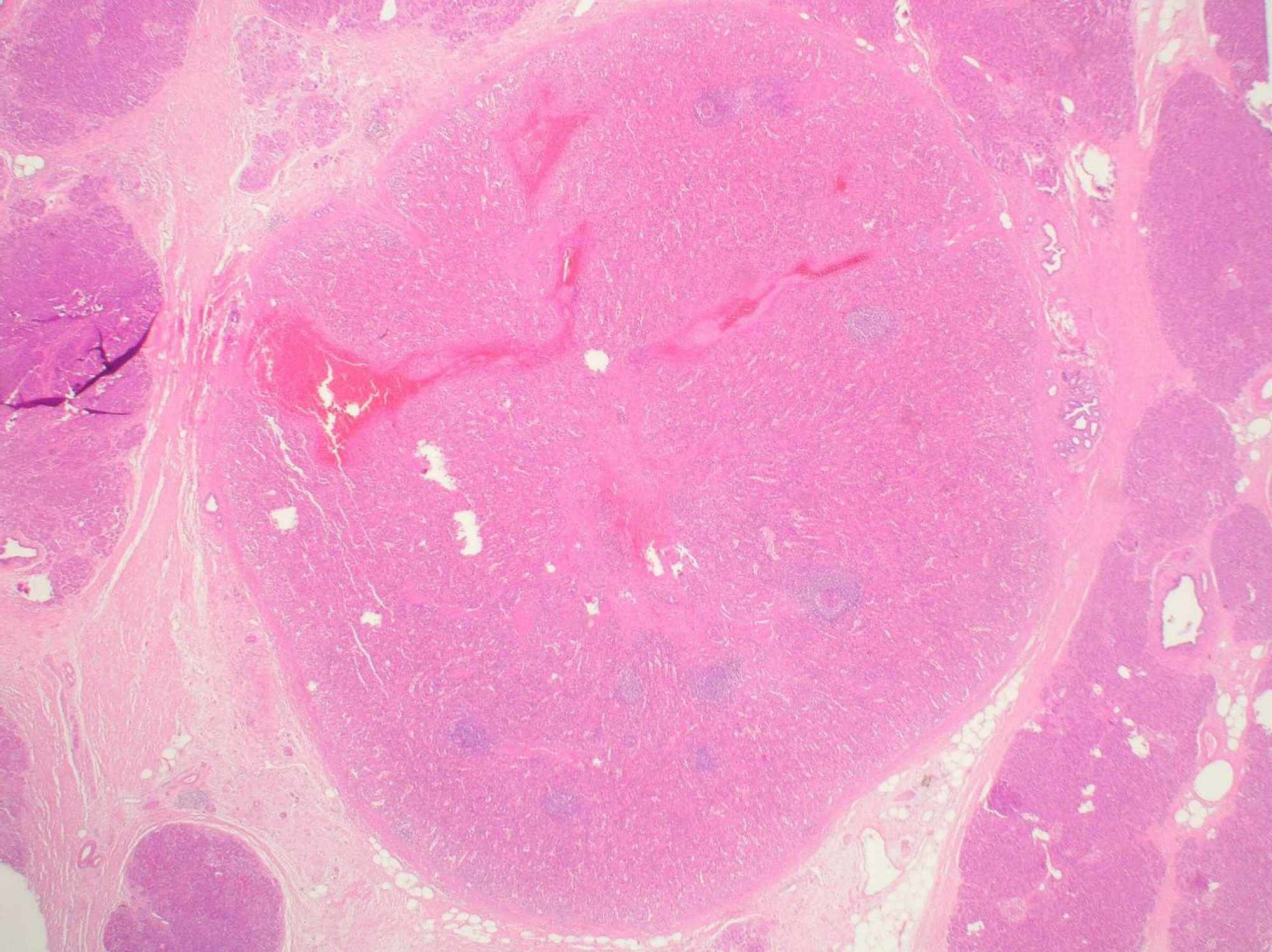
Regular structured splenic tissue (center) encircled from pancreatic tissue with endocrine and exocrine fractions and exportive ducts with light fibrosis Hematoxylin and eosin staining (magnification x25)

**Figure 4:**
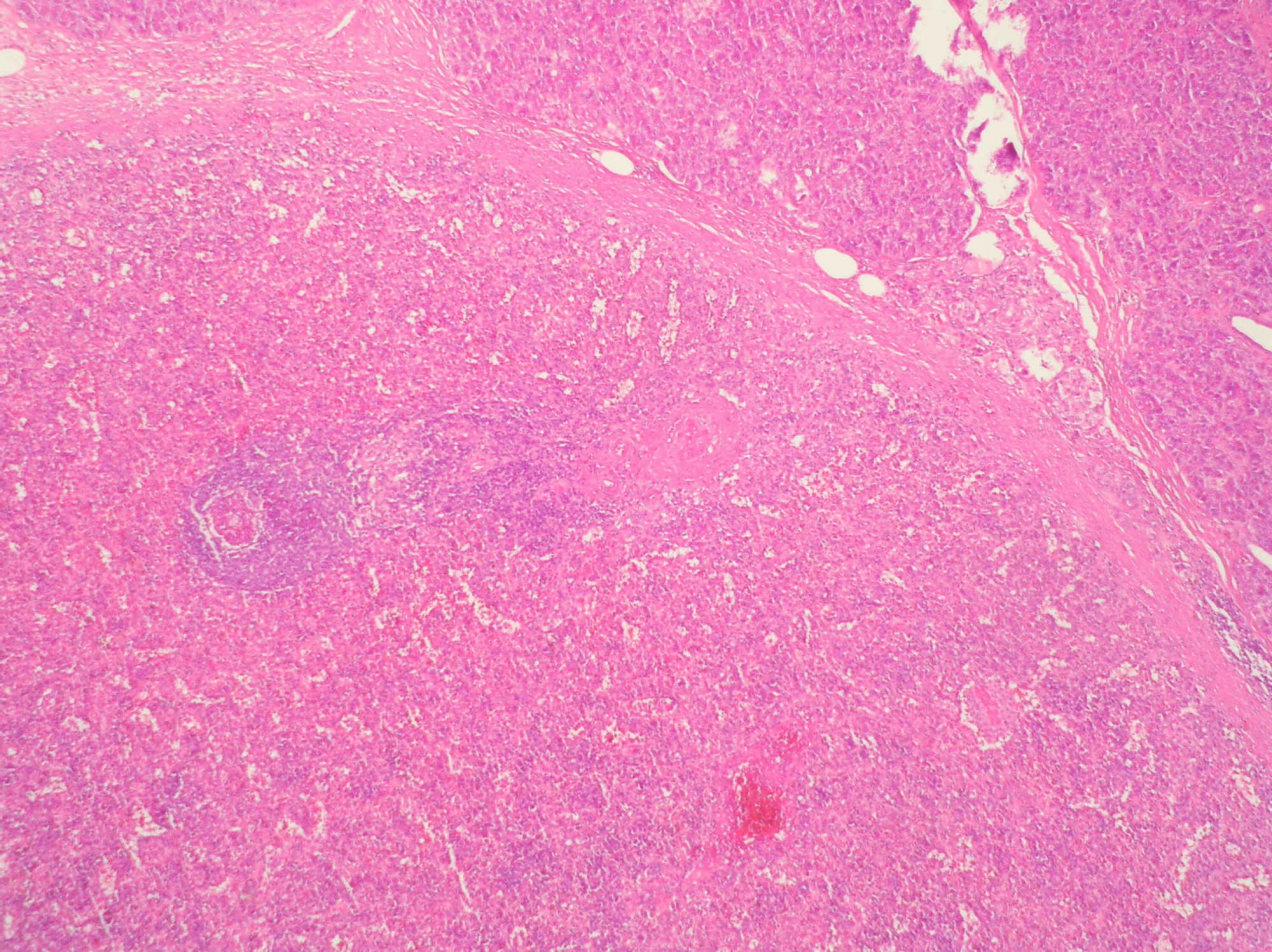
Encapsulated splenic tissue well differentiated from the adjacent pancreas Hematoxylin and eosin staining (magnification x100)

CEA and CA 19-9 were within normal laboratory ranges. After an unsuccessful endoscopic trans-gastric drainage and subsequent abscess formation in the pancreatic tail, the patient was treated with a distal pancreatectomy. Histopathological examination of the specimen showed a 2-cm auxiliary spleen within the pancreatic tail (Figures 3, 4). The postoperative course was uneventful and the patient was discharged one week after the operation.

## Discussion

Accessory spleens are small nodules of ectopic splenic tissue. They likely represent remnants of tissue along the embryologic splenic migratory pathway from the midline to its final location of the left side of the abdomen at the level of the 9th-11th ribs. In contrast, the entity of splenosis represents the presence of heterotopic foci of splenic tissue, occurring through autotransplantation after splenic trauma or surgery. Autopsy studies report a 10% incidence of accessory spleens in the general population [4]. Although 80% of accessory spleens are located within the splenic hilum or adjacent to the tail of the pancreas, they may also be found anywhere along the splenic vessels, in the gastrosplenic ligament, the splenorenal ligament, the walls of the stomach or intestines, the pancreatic tail, the greater omentum, the mesentery, or the gonads and their path of descent. Intra and peripancreatic spleens represent only 10-16% of all accessory spleens. Their typical dimension is approximately 1 cm in diameter, but sizes ranging from a few millimeters up to 2-3 cm are not uncommon [3,4].

Intrapancreatic accessory spleens usually represent an incidental finding, but obtaining an accurate diagnosis is often challenging. More specifically, differentiating them from hypervascular pancreatic neoplasms (primary or metastatic), complicated cystic formations, or splenic hilar lymphadenopathy might be quite difficult. A routine laboratory test in most cases do not reveal any biochemical abnormalities. Tumor markers such as CEA and CA 19-9 are usually within normal ranges. Biochemical tests for the identification of possible neuroendocrine or genetic markers are very expensive, while their results usually fall within normal ranges.

Abdominal ultrasonography depicts intrapancreatic accessory spleens as well-defined masses with a lower echogenicity than that of the surrounding pancreas. The Doppler effect may reveal direct blood flow from/to the splenic vessels to/from the intrapancreatic accessory spleen [5]. According to Ota et.al., its diagnostic capacity may be further increased by using contrast agent enhancement [6]. Although cost-effective, ultrasonography is operator-dependent and lacks sensitivity, especially in obese patients, in whom the pancreatic tail is hard to visualize.

Most accessory spleens have a characteristic configuration on a CT scan, appearing as well-marginated round masses with homogeneity similar to that of the spleen. As described by Hayward et al., intrapancreatic accessory spleens can be suspected when an intrapancreatic mass enhances in a manner identical to that of the spleen [7]. Similarly, accessory spleens may be differentiated from metastatic lesions or splenic hilar lymphadenopathy, as their enhancement is similar to that of the spleen. However, according to Mortelé et al., almost one out of three accessory spleens smaller than 2 cm is interpreted as a hypogenic lesion [8]. Another diagnostic difficulty may occur when trying to differentiate accessory spleens from hypervascular pancreatic neoplasms such as islet tumors or hypervascular metastases. Although thin (5 mm or less) collimation may help resolve the problem of hypogenicity, the differentiation from other tumors remains difficult.

MRI demonstrates similar T1 and T2 signal intensity between the spleen and accessory spleens. In most cases, both have identical density and have been described as solid masses. MRI can also show cystic degeneration and hemorrhage, both of which are more frequent in malignant cases [9,10]. Nuclear scintigraphy using technetium-99m (Tc-99m), radioablated heat-damaged red blood cells, and indium (In)-111 labeled autologous platelets can provide further information in instances of inconclusive CT or MRI findings [11]. Schreiner et al. successfully diagnosed accessory spleens with endoscopic ultrasound (EUS)-guided FNA and CD8 immunostaining of splenic sinus endothelial cells [12]. Although small, this series introduced a promising minimally invasive diagnostic approach with few potential complications.

Although the surgical resection of an intrapancreatic spleen is generally not indicated, the diagnostic difficulty in excluding a pancreatic malignancy often leads to a distal pancreatectomy. Additional reasons for surgical intervention include complications or spleen-related hemato-oncological conditions such as immune thrombocytopenia (ITP). In cases of surgical indication, possible modalities include open, laparoscopic, or even robotic approach. The choice of the most suitable modality depends on the patient’s characteristics, the location and size of the lesion, and prior experience of the surgical team. Furthermore, the decision whether to perform a simultaneous splenectomy (if prior not performed) or not is taken based on lesion's relationship to main splenic vessels and the suspicion index for malignancy.

## Conclusions

Intrapancreatic accessory spleens are uncommon entities, and their surgical resection is usually not indicated except in symptomatic cases and cases requiring splenectomy for hemato-oncological indications or ITP. Distal pancreatectomy with or without splenectomy may be complicated by subphrenic abscess formation and pancreatic duct leak (incidence of up to 10% and 20%, respectively). The presence of an intrapancreatic accessory spleen should be part of the differential diagnosis of pancreatic lesions in order to avoid unnecessary surgical interventions.
